# Acute Kidney Injury Associated With Red Yeast Rice (Beni-kōji) Supplement: A Report of Two Cases

**DOI:** 10.1016/j.xkme.2024.100908

**Published:** 2024-09-19

**Authors:** Kiyotaka Uchiyama, Masako Otani, Naoki Chigusa, Kazuya Sugita, Ryosuke Matsuoka, Koji Hosoya, Mina Komuta, Jun Ito, Naoki Washida

**Affiliations:** 1 Department of Nephrology, International University of Health and Welfare Narita Hospital, Chiba, Japan; 2 Department of Pathology, International University of Health and Welfare Narita Hospital, Chiba, Japan; 3 Department of Pathology, International University of Health and Welfare Mita Hospital, Tokyo, Japan; 4 Department of Nephrology, International University of Health and Welfare Hospital, Tochigi, Japan

**Keywords:** Red yeast rice, kidney injury, tubular injury, Fanconi syndrome, foods with functional claims

## Abstract

Numerous health concerns, primarily kidney injury, have been reported with the use of Beni-kōji CholesteHelp, a functional food containing red yeast rice. Here, we describe 2 cases of kidney injury caused by beni-kōji. The first case had normal kidney function before consuming the product. After several months of use, she developed hypertension. After 6 months of supplement consumption, her estimated glomerular filtration rate (eGFR) dropped to 22.5 mL/min/1.73 m^2^. A spot urine sample showed a urinary protein-to-creatinine ratio of 2.03 g/g, leading to the diagnosis of Fanconi syndrome. Kidney biopsy showed tubular degeneration. Thirty-five days after discontinuing the supplement, proteinuria resolved and the eGFR returned to baseline level. The second case, who had diabetes and normal kidney function, experienced severe kidney injury (eGFR, 3.5 mL/min/1.73 m^2^) after 4 months of Beni-kōji CholesteHelp use. He required hemodialysis for >2 weeks but recovered kidney function after the product was discontinued. Kidney biopsy showed tubular injury similar to the first case and glomeruli changes consistent with diabetic nephropathy. These cases indicate that beni-kōji use is associated with tubular toxicity. Further studies are required to identify the precise etiology and mechanism of kidney injury.

## Introduction

Several drugs can cause kidney damage, such as over-the-counter medicines and foods with functional claims.^1^ Red yeast rice (RYR), called “*beni-kōji*” in Japanese, has been widely used as a dietary supplement or functional food in Asia and Europe. It lowers low-density lipoprotein cholesterol levels by producing monacolin K, which is synonymous with lovastatin.^2^ Beni-kōji CholesteHelp, developed and marketed by Kobayashi Pharmaceuticals, is a food with functional claims of lowering low-density lipoprotein cholesterol levels that contains RYR. However, this product has caused several health issues, mainly kidney injury, need for hospitalization, and even death, prompting product recall (https://www.japantimes.co.jp/news/2024/04/09/japan/science-health/health-ministry-beni-koji-presser/). Here, we have reported 2 cases of severe kidney injury with tubular damage that developed after consumption of Beni-kōji CholesteHelp.

### Case Report

**Fig 1:**
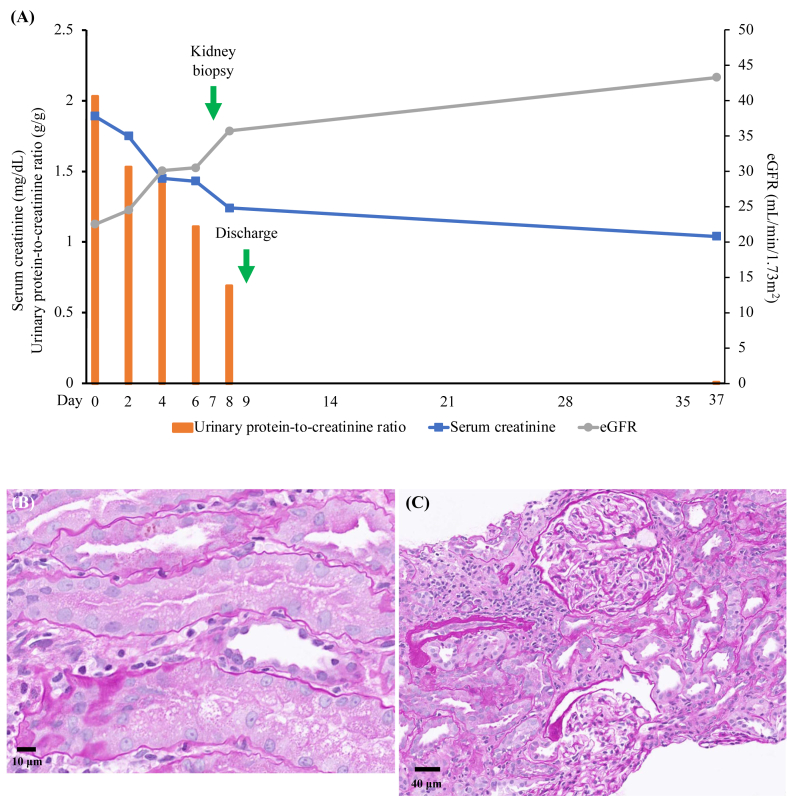
(A) Clinical course and (B-C) micrographs of kidney biopsy specimen of case 1. (A) Clinical course. (B) Light microscopy of periodic acid–Schiff-stained sections of the biopsy specimen demonstrating prominent tubular degeneration and some tubular atrophy. Swelling, flattening, and bleb formation of tubular epithelial cells can be observed. Tubulitis is not prominent, and interstitial inflammatory cell infiltration is insignificant. The inflammatory cells are predominantly lymphocytes and plasma cells, with some eosinophils. (C) Light microscopy of periodic acid–Schiff-stained sections of the biopsy specimen demonstrating normal glomeruli. Abbreviations: eGFR, estimated glomerular filtration rate.

Case 1 was a 56-year-old woman with no significant past medical history. She started consuming 3 tablets/day (recommended dosage) of Beni-kōji CholesteHelp in June 2023. She had been regularly visiting a neurosurgeon because of a family history of stroke. In July 2023, her baseline serum creatinine (SCr) and estimated glomerular filtration rate (eGFR) levels were within normal limits (0.7 mg/dL and 67.1 mL/min/1.73 m^2^, respectively). In November 2023, she developed hypertension, with a systolic blood pressure of 160 mm Hg. On December 21, 2023 (day 0), during a routine visit to the neurosurgeon, blood reports showed an elevated SCr level (1.89 mg/dL) and low eGFR level (22.5 mL/min/1.73 m^2^) (Fig 1A). In addition, she had marked proteinuria (dipstick, 1+), increased urinary protein-to-creatinine ratio (UPCR, 2.03 g/g), glucosuria (dipstick 4+), hypouricemia (1.9 mg/dL), hypokalemia (3.5 mmol/L), hypophosphatemia (1.9 mg/dL), and metabolic acidosis (pH 7.207) with a low serum bicarbonate level (20.4 mmol/L). These findings were suggestive of Fanconi syndrome. Creatinine kinase levels were within normal limits (77 U/L), ruling out rhabdomyolysis. Thus, Beni-kōji CholesteHelp was discontinued. At the first visit to our nephrology department (day 3), SCr level (1.75 mg/dL), eGFR (24.5 mL/min/1.73 m^2^), and UPCR (1.53 g/g) had improved. However, the urinary albumin-to-creatinine ratio was 252.6 mg/g, and the urinary β2-microglobulin-to-creatinine ratio was 90 mg/g. The urinary nonalbumin protein level was high (1,277.4 mg/g), and could not be explained by the β2-microglobulin levels.^3^ This indicated that amino acids were present in the urine, based on the previous diagnosis of Fanconi syndrome. However, the amino acids were not analyzed. Abdominal computed tomography did not demonstrate any evidence of renal swelling or atrophy. Kidney biopsy, performed on day 8, showed 7 glomeruli. One glomerulus was globally sclerosed, and the others did not exhibit any remarkable findings. Tubular degeneration, particularly in proximal tubules, was prominent, but tubulitis was not observed. Interstitial inflammatory cell infiltration was present but was not significant. The inflammatory cells were predominantly lymphocytes and plasma cells, with some eosinophils (Figs 1B and C). On day 9, SCr level reduced to 1.24 mg/dL, eGFR increased to 35.7 mL/min/1.73 m^2^, and proteinuria reduced, and UPCR was 0.69 g/g. Therefore, prednisolone was not administered. On day 35, SCr and eGFR levels were 1.04 mg/dL and 43.3 mL/min/1.73 m^2^, respectively, and proteinuria had subsided.

**Fig 2:**
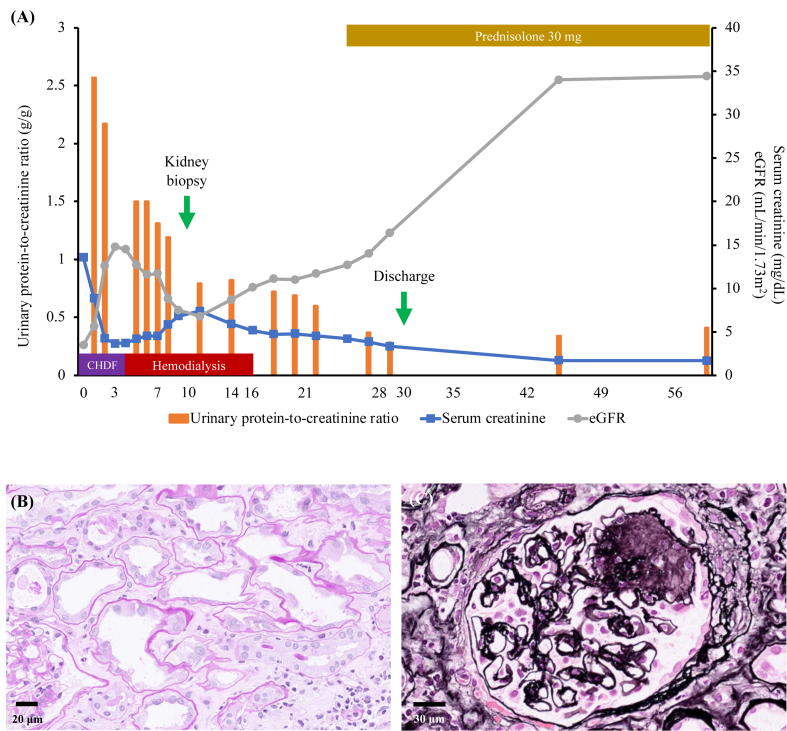
(A) Clinical course and (B-C) micrographs of kidney biopsy specimen of case 2. (A) Clinical course. Serum creatinine levels were obtained during the CHDF session and just before the session when he was on intermittent hemodialysis. (B) Light microscopy of periodic acid–Schiff-stained sections of the biopsy specimen showing marked tubular degeneration. Tubular epithelial cells are swollen and flattened, and cellular debris can be observed in some tubular lumens. Inflammatory cell infiltration of the tubulointerstitium is insignificant. The inflammatory cells are mainly lymphocytes, plasma cells, and neutrocytes. (C) Light microscopy of a periodic acid-methenamine silver-stained biopsy specimen showing nodular glomerulosclerosis, a typical finding of diabetic nephropathy. Abbreviations: CHDF, continuous hemodiafiltration; eGFR, estimated glomerular filtration rate.

Case 2 was a 56-year-old-man with a 13-year history of diabetes mellitus (DM). He was being treated with sitagliptin (50 mg/d), dapagliflozin (5 mg/d), glimepiride (0.5 mg/d), and metformin (1,500 mg/d). His glycated hemoglobin (A1c) level was controlled at 6.5%-7.3%, and no signs of diabetic retinopathy were present. He started consuming 3 tablets per day of Beni-kōji CholesteHelp in early September 2023. A blood analysis for regular DM checkup on November 9, 2023, showed normal kidney function (baseline SCr, 0.93 mg/dL and eGFR, 66.2 mL/min/1.73 m^2^). On January 19, 2024, his appetite decreased, and Beni-kōji CholesteHelp was discontinued. The following day, he developed nausea and vomiting. Because he had difficulty in consuming his oral medications since January 22, 2024 and was unable to speak from midnight the following day, he was transported by an ambulance to an emergency department on January 24, 2024. On arrival (day 0), his kidney function was severely impaired (SCr level, 13.55 mg/dL) (Fig 2A). In addition, lactic acidosis (pH < 6.75) and a high serum lactate level (15 mmol/L) was detected on admission. This was considered to be caused by metformin accumulation because of severe kidney injury. Abdominal computed tomography revealed normal kidneys and no evidence of hydronephrosis. Creatinine kinase level was normal (122 U/L). Continuous hemodiafiltration was administered up to day 3. Thereafter, because of complicated hypotension, intermittent hemodialysis was performed up to day 17. A detailed urinalysis was performed on day 3, which showed proteinuria (dipstick, 1+), glucosuria (dipstick, 4+), and hematuria (dipstick, 3+) with 5-9 erythrocytes per high-power field. Microscopic hematuria may have been caused by Foley’s catheter insertion. The UPCR, urinary albumin-to-creatinine ratio, and urinary β2-microglobulin-to-creatinine ratio were 2.17 g/g, 637.5 mg/g, and 70 mg/g, respectively, and the urinary nonalbumin protein was high (1,532.5 mg/g). These findings indicated the presence of amino acids in the urine, similar to the conclusion drawn in case 1. Therefore, although hypokalemia, hypophosphatemia, and hypouricemia were not remarkable because of severe kidney injury, the presence of Fanconi syndrome could not be ruled out. A kidney biopsy was performed on day 10, which showed 22 glomeruli. Of these, 3 glomeruli were globally sclerosed and 4 demonstrated nodular glomerulosclerosis, which indicated diabetic nephropathy. Severe tubular degeneration with vacuolation or flattening of tubular epithelial cells was observed, with minimal inflammatory cell infiltration of the tubulointerstitium. The inflammatory cells included lymphocytes, plasma cells, eosinophils, and neutrophils (Figs 2B and C). Although he was anuric on day 0, his daily urinary volume increased to 590 mL on day 2, 1,260 mL on day 5, and 2,400 mL on day 8. His prehemodialysis SCr levels began to improve on day 14. On day 16, hemodialysis was discontinued. On day 25, although his SCr level continued to decline, prednisolone was initiated (30 mg/d). The UPCR also continued to decrease during the hospitalization. He was discharged on day 30 with a SCr level of 3.32 mg/dL. During an outpatient clinic visit after discharge, the SCr had further decreased to 1.69 mg/dL.

## Discussion

Although reports of Beni-kōji CholesteHelp allegedly causing kidney injury exist, mainly in the mass media, it has become a social problem, mainly in Japan. This report provides detailed clinical courses and renal pathology of 2 cases who developed kidney injury after consuming Beni-kōji CholesteHelp. In both patients, although there was no decline in kidney function in the initial 2 months, they became symptomatic with a marked decreased in kidney function 4-5 months after continuous consumption of Beni-kōji CholesteHelp. This suggests that accumulation of some substance in Beni-kōji CholesteHelp had caused direct tubular injury because of toxicity to various cellular functions; an allergic T-cell mediated immune response had not developed.^1^ A crystalline-related kidney injury because of the precipitation of some substance within the distal tubular lumens was ruled out on histopathological examination. Although case 2 required hemodialysis for some time, discontinuation of Beni-kōji CholesteHelp alone resulted in the spontaneous recovery of kidney function in both cases. Although prednisolone was administered to case 2, its effect remains unknown because it was initiated during spontaneous kidney function improvement.

An interim report, as of March 31, 2024, on the nationwide survey issued by the Japanese Society of Nephrology, shows that most of the 47 patients with suspected kidney injury because of the products demonstrated signs of Fanconi syndrome, such as proteinuria, hypokalemia, hypophosphatemia, hypouricemia, metabolic acidosis, and glucosuria.^4^ In patients in whom kidney biopsies were performed, tubular damage was reportedly remarkable, and the clinical and pathologic findings were consistent with those of our patients. However, the causative substance is yet to be identified (as of April 3, 2024). RYR is rice fermented using the fungus Monascus. Some Monascus strains reportedly produce the mycotoxin citrinin,^5^ which is nephrotoxic and mainly causes tubular damage.^6^ However, the R&D Center at Kobayashi Pharmaceutical Co investigated the diversity of secondary metabolite production in 3 *Monascus* species at both the metabolome level, using LC-MS analysis, and at the genome level.^7^ They reported that the citrinin biosynthetic gene were incomplete in *Monascus pilosus* (NBRC4520) and *Monascus ruber* (NBRC4483), and thus citrinin production is blocked in these species. Beni-kōji CholesteHelp uses these species, and the company has reported that citrinin was not detected in the products. Furthermore, although there are reports of RYR, such as that in statin, causing rhabdomyolysis-induced acute kidney injury,^8^ the association between RYR itself and kidney injury is considered weak.^9^ However, Kobayashi Pharmaceuticals found puberulic acid, a natural cytotoxic compound made from blue mold,^10^ in Beni-kōji CholesteHelp. However, it remains to be determined why puberulic acid was present in the product and whether it was the cause for the health problems.

In conclusion, 2 case series of kidney injury associated with Beni-kōji CholesteHelp are reported. These cases suggest direct tubular toxicity associated with Beni-kōji supplement use. Further studies are needed to identify the precise etiology and mechanism of kidney injury. We hope that this report will contribute to improving the condition of patients with Beni-kōji CholesteHelp-induced kidney injury and elucidating its pathophysiology.
